# Supplementary material for: Tandem hnRNP A1 RNA recognition motifs act in concert to repress the splicing of survival motor neuron exon 7
Source: eLife. 2017 Jun 26;6:e25736. doi: 10.7554/eLife.25736 (PMC5503513; doi:10.7554/eLife.25736)
Supplement: Table 4—source data 1. — DOI: http://dx.doi.org/10.7554/eLife.25736.013 [file elife-25736-table4-data1.docx]

**Table 4 – source data 1: ITC measurements of protein variants with 5´-UUAGUU-3´.**

Isothermal titration calorimetry monitoring the binding of single RRMs or UP1 RRM mutants to UUAGUU. The displayed titrations correspond to data shown in Table 4.

**(A**) RRM1 + UUAGUU

(**B**) UP1-R1r2 + UUAGUU

(**C**) RRM2 + UUAGUU

(**D**) UP1-r1R2 + UUAGUU
